# P450_BM3_ fused to phosphite dehydrogenase allows phosphite-driven selective oxidations

**DOI:** 10.1007/s00253-016-7993-7

**Published:** 2016-11-29

**Authors:** Nina Beyer, Justyna K. Kulig, Anette Bartsch, Martin A. Hayes, Dick B. Janssen, Marco W. Fraaije

**Affiliations:** 1 Department of Biochemistry, Groningen Biomolecular Sciences and Biotechnology Institute, University of Groningen, Nijenborgh 4, 9747 AG Groningen, The Netherlands; 2 Cardiovascular and Metabolic Diseases, Innovative Medicines and Early Development Biotech Unit, AstraZeneca, Pepparedsleden 1, Mölndal, 43183 Sweden

**Keywords:** Drug metabolites, Enzyme catalysis, Fatty acids, NADPH regeneration, Oxidation, Protein engineering

## Abstract

To facilitate the wider application of the NADPH-dependent P450_BM3,_ we fused the monooxygenase with a phosphite dehydrogenase (PTDH). The resulting monooxygenase-dehydrogenase fusion enzyme acts as a self-sufficient bifunctional catalyst, accepting phosphite as a cheap electron donor for the regeneration of NADPH.

The well-expressed fusion enzyme was purified and analyzed in comparison to the parent enzymes. Using lauric acid as substrate for P450_BM3_, it was found that the fusion enzyme had similar substrate affinity and hydroxylation selectivity while it displayed a significantly higher activity than the non-fused monooxygenase. Phosphite-driven conversions of lauric acid at restricted NADPH concentrations confirmed multiple turnovers of the cofactor. Interestingly, both the fusion enzyme and the native P450_BM3_ displayed enzyme concentration dependent activity and the fused enzyme reached optimal activity at a lower enzyme concentration. This suggests that the fusion enzyme has an improved tendency to form functional oligomers.

To explore the constructed phosphite-driven P450_BM3_ as a biocatalyst, conversions of the drug compounds omeprazole and rosiglitazone were performed. PTDH-P450_BM3_ driven by phosphite was found to be more efficient in terms of total turnover when compared with P450_BM3_ driven by NADPH. The results suggest that PTDH-P450_BM3_ is an attractive system for use in biocatalytic and drug metabolism studies.

**Electronic supplementary material:**

The online version of this article (doi:10.1007/s00253-016-7993-7) contains supplementary material, which is available to authorized users.

## Introduction

Cytochrome P450 monooxygenases (P450s) are versatile enzymes that catalyze a broad range of hydroxylation, epoxidation, sulfoxidation, deamination, and dehalogenation reactions and are attractive for applied biocatalysis (Sono et al. 1996; Bernhardt 2006). In vivo, the main functions include steroid hydroxylation, fatty acid hydroxylation, and xenobiotic detoxification, which consequently makes P450s of interest to the pharmaceutical industry (Gillam and Hayes 2013; Munro et al. 2013). Similar to other monooxygenases, catalysis requires a suitable substrate, dioxygen, and two electrons to be fed into the catalytic reaction cycle (Meunier et al. 2004). The electrons are transferred to the heme from a reducing cofactor via electron transfer proteins, which may be domains fused to the monooxygenase component or may occur as separate proteins transferring electrons in transient assemblies (Hannemann et al. 2007). The need for auxiliary redox proteins presents an obstacle for the efficient application of P450s because (a) complex cofactors have to be integrated into different proteins or protein domains to obtain a functional catalyst and (b) participating enzymes need to match each other in terms of concentration, stability, affinity, and activity to achieve maximal turnover. In addition, the dependency on NAD(P)H, slow electron transport, and coupling efficiency can limit activity (Lundemo and Woodley 2015).

The highly active bacterial flavocytochrome P450_BM3_ (CYP102A1 from *Bacillus megaterium*, EC 1.14.14.1) naturally circumvents some of these limitations. As a single polypeptide P450 (class VIII), it has an FAD- and FMN-containing NADPH-cytochrome P450 reductase (BMR domain) fused to the C-terminus of the P450 domain in a 119 kDa protein, that is active as a dimer (Ruettinger et al. 1989; Neeli et al. 2005). The enzyme can be solubly expressed in good yields in heterologous hosts such as *Escherichia coli*. It is one of the few P450s with reported activities above 1 s^−1^, which is partly due to rapid electron transfer through the BMR domain (Munro et al. 1996; Munro et al. 2002). The enzyme efficiently hydroxylates medium- to long-chain fatty acids. Arachidonic acid was reported to be converted with the highest activity (285 s^−1^). Low uncoupling rates of approx. 3.8% were determined when using laurate as substrate (Noble et al. 1999). After it was shown that the substrate spectrum could be modulated by various mutations, P450_BM3_ was widely used as a surrogate for class II mammalian P450s. It also is used as a model P450 in mechanistic studies because of easy expression, high activity, and the availability of crystal structures of the separate domains. Whitehouse et al. reviewed recent progress on this enzyme and describe numerous mutants accepting non-natural substrates; many of them are bulky molecules like drugs and related compounds (Whitehouse et al. 2012).

The regeneration of NADPH, which is stoichiometrically consumed in the monooxygenation reaction, remains a major challenge for the application of P450s. Several regeneration approaches have been investigated, of which the enzymatic regeneration of the cofactor is most commonly applied. One possibility is the use of dehydrogenases; popular examples being glucose-6-phosphate dehydrogenase (Ahmed et al. 1999; Falck et al. 2001; Volz et al. 2002; Murataliev et al. 2004; Chen et al. 2008), glucose dehydrogenase (Sulistyaningdyah et al. 2005; Schewe et al. 2008; Schewe et al. 2009), isocitrate dehydrogenase (Capdevila et al. 1996; Schwaneberg et al. 2001; Peters et al. 2003; Landwehr et al. 2006), and formate dehydrogenase (Maurer et al. 2003; Kühnel et al. 2007). Phosphite dehydrogenase (PTDH, EC 1.20.1.1) is one of the most cost efficient options; it regenerates NADPH while oxidizing cheap phosphite to phosphate with the simultaneous reduction of NADP^+^. The large change in energy of this reaction makes it thermodynamically almost irreversible and only one inhibitor (sulfite) has been described to date (Costas et al. 2001; Woodyer et al. 2003; Relyea and van der Donk 2005).

The recycling of NADPH with phosphite has been reported for fusions of PTDH with several Baeyer-Villiger monooxygenases (BVMOs) (Torres Pazmiño et al. 2009). A codon-optimized gene was used for expression of PTDH-BVMO fusions in *E. coli*. The employed PTDH contained 18 mutations which led to enhanced activity towards NADP^+^, soluble expression, and stability (Johannes et al. 2007). Watanabe et al. also reported a fusion of PTDH to a Pdx-PCNA2 construct which functioned as the “PCNA-utilized protein complex of P450cam and its two electron transfer-related proteins” (PUPPET) for the regeneration of NADH (Watanabe et al. 2013).

**Scheme 1:**
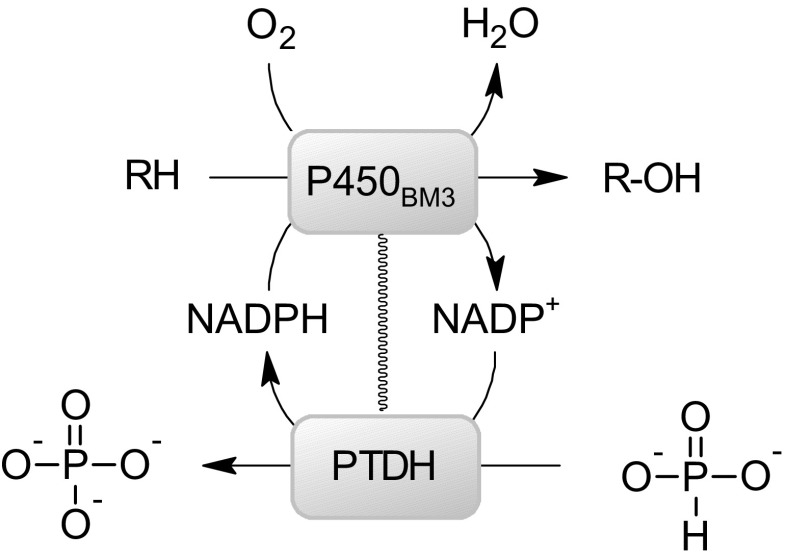
Schematic model of PTDH coupled to P450_BM3_ facilitating phosphite-driven cofactor recycling

To facilitate the application of P450_BM3_ as a practical biocatalyst, we present here a fusion of P450_BM3_ to the optimized variant of PTDH for cofactor recycling: PTDH-P450_BM3_. The turnover of NADP^+^ to NADPH by the fused PTDH domain should enable the use of phosphite as a cheap reductant for performing P450_BM3_-catalyzed oxygenations with phosphate as a byproduct (Scheme 1). The fusion protein is compared to the regeneration system with the free PTDH and P450_BM3_ and the P450_BM3_ without cofactor regeneration system.

## Materials and methods

### Materials

Unless stated otherwise, all chemicals and enzymes were obtained from Sigma-Aldrich (Zwijndrecht, The Netherlands), Oriental Yeast Co. (Tokyo, Japan), New England Biolabs (Leiden, The Netherlands), and Agilent (Santa Clara, USA) and used without further purification. Isopropyl-1-thio-β-D-galactopyranoside (IPTG) was ordered at Iris Biotech GmBH (Marktredwitz, Germany). Oligonucleotide primers were obtained from Sigma Genosys. DNA sequencing was performed at GATC Biotech AG (Konstanz, Germany).

### Bacterial strains, plasmids, and cloning

*E. coli* TOP10 (Invitrogen, Carlsbad, CA, USA) was used for genetic manipulations. For expression, a pBAD vector or a modified pBAD vector with an *Nco*І restriction site replacing *Nde*І were used. The plasmid pPTDH in which the codon-optimized ptxD gene is fused to an N-terminal histidine tag codes the 18-fold mutated PTDH without a fused monooxygenase. The vector pJOE-P450_BM3_ encoding the wild-type gene *cyp102A1* was a kind gift from Prof. B. Hauer (University of Stuttgart) (Scheps et al. 2013). For the creation of the fusion enzyme, the vector pCRE2-PAMO was used as a template. It carries the 18-fold mutant of the phosphite dehydrogenase from *Pseudomonas stutzeri* WM88 with N-terminal histidine tag and the *pamO* gene in fusion (Torres Pazmiño et al. 2009). The fusion construct pCre2-P450_BM3_ was made implementing the In-Fusion technique from Clontech, resulting in the sequence coding for the fusion construct PTDH-P450_BM3_ (KX768143). Using primers with overhangs matching the expression vector, *cyp102A1* encoding P450_BM3_ was amplified via PCR using pJOE-P450_BM3_ as template and then hybridized with the respective expression vector, following the recommendations of the manufacturer. For comparison studies with the unfused enzyme, *cyp102A1* was subcloned into pBAD. Implementing primers with an overhang matching the target vector, *cyp102A1* was amplified via a PCR reaction. The PCR product was purified and utilized as a primer for a second PCR reaction, subcloning the gene encoding P450_BM3_ into the respective expression vector, yielding pBAD-P450_BM3_.

### Screen for soluble enzyme expression

*E. coli* TOP10, BL21 (DE3), C43 (DE3) and SHuffle cells harboring pCre2-P450_BM3_ were pre-grown overnight at 37 °C in ampicillin-supplemented LB medium (5 mL; 50 μg/mL). TB-medium (50 mL, ampicillin-supplemented) in a non-baffled glass shaking flask (250 mL) was inoculated with an OD_600_ of 0.08 and incubated at 37 °C and 180 rpm. After an OD_600_ of 0.75–0.85 was reached, expression of PTDH-P450_BM3_ was induced by the addition of 0.02% arabinose (final concentration). The cells were incubated for 25 h at 30 °C and 135 rpm. Cells were harvested by centrifugation (20 min, 8800×*g*, 4 °C), washed with ice-cold sodium phosphate buffer (50 mM, pH 7.5) and frozen (−20 °C) until cell lysis by sonication. The cell debris was separated by centrifugation and the content of PTDH-P450_BM3_ in the cell-free extract was investigated via CO-difference spectroscopy. For the optimization of the arabinose concentration and the temperature for enzyme expression, TB-medium (2.5 mL) in 24-square deep well plates with the sandwich cover system from enzyscreen®, was inoculated with a preculture of *E. coli* TOP10, harboring pCre2-P450_BM3_ (5 μL). Cultures were grown at 17 °C (48 h), 24 °C (32 h), 30 °C (16 h), and 37 °C (16 h) and expression was induced directly after inoculation with 0% (as control), 0.002%, 0.02%, and 0.2% of final arabinose. Cells were harvested via centrifugation and cell pellets were frozen (−20 °C, 16 h). Cell lysis was performed enzymatically (200 μL lysis buffer consisting of 2 mg/mL lysozyme, 0.1 mg/mL DNasel, 5 mM MgSO_4_ in 50 mM Tris/HCl (pH 8)) and with a freeze-and-thaw cycle (plate was shock frozen in liquid nitrogen and incubated in a shaker at 30 °C for 1 h). Samples were taken and the cell debris was separated from the soluble fraction by centrifugation. Both the lyzed cell and the soluble fraction were analyzed for expression of PTDH-P450_BM3_ via SDS-PAGE.

### Enzyme expression

*E. coli* TOP10 cells harboring pBAD vectors encoding the respective P450_BM3_ variants were pre-grown overnight (37 °C) in 5 mL ampicillin-supplemented LB medium (50 mg/mL). Four hundred milliliters of TB-medium (ampicillin-supplemented) in non-baffled glass shaking flask (2 L) were inoculated with an OD_600_ of 0.08 and incubated at 37 °C and 180 rpm. Expression was induced at an OD_600_ of 0.7–0.8 with arabinose (0.02% *w*/*v*) and 5-aminolevulinic acid was added (0.5 mM). After induction, the cells were incubated (17 °C, 180 rpm) over 44 h, harvested via centrifugation, washed once with ice-cold sodium phosphate buffer (50 mM, pH 7.5), and were stored until further use (−20 °C).

The PTDH was expressed as described before by Dudek et al. (Dudek et al. 2013). *E. coli* TOP10 cells were transformed with the plasmid and grown overnight at 37 °C in 5 mL of LB supplemented with ampicillin (50 μg/mL). The next day cells were diluted (1:100) in TB supplemented with ampicillin (50 μg/mL) and arabinose (0.02% *w*/*v*) and incubated for 16 h at 30 °C. Cells were harvested via centrifugation, washed once with ice-cold sodium phosphate buffer (50 mM, pH 7.5), and were stored until further use (−20 °C).

### Enzyme purification

For purification, the frozen cells were resuspended in sodium phosphate buffer (50 mM, pH 7.5, optionally supplemented with DNase) at RT and disrupted via sonication on ice. The resulting crude extract was centrifuged and the supernatant/cell-free extract (CFE) diluted to a protein concentration of ∼20 mg/mL. The diluted CFE was then precipitated at an ammonium sulfate (AS) concentration of 55% (*w*/*v*) and collected by centrifugation (18,600×*g*, 15 min, 4 °C). The resulting pellet was resuspended in sodium phosphate buffer (50 mM, 500 mM NaCl pH 7.5) and subjected to an affinity chromatography purification on a 5 mL HF HisTrap column on an Äkta purifier (GE Healthcare). Fractionated elution was carried out with imidazole (250 mM). The buffer was removed by a second AS precipitation with 65% AS. The resulting pellet was resuspended in ice-cold Tris/HCl. After the addition of sucrose (20 mg/mL) for stabilization during storage, the enzyme was shock frozen in liquid nitrogen and stored at −80 °C (van Beek et al. 2015).

The PTDH was purified using Ni Sepharose High Performance (GE Healthcare) as described before (Dudek et al. 2013). Ten percent glycerol (*v*/*v*) was added to the purified enzyme, which was shock frozen and stored at −20 °C.

### Concentration determination

Concentrations of P450_BM3_ and PTDH-P450_BM3_ were determined by measuring CO-difference spectra (Omura and Sato 1964). After the addition of sodium dithionite (spatula tip), the enzyme solution was incubated (5 min) and a baseline spectrum from 400 to 500 nm was recorded. The samples were then bubbled with carbon monoxide (∼60 s) and incubated for 10 min before the UV spectrum from 400 to 500 nm was recorded. After subtraction of the baseline spectrum, the amount of P450 was calculated based on the maximum absorbance at 450 nm minus the absorption at 490 nm (ε_450nm_ = 91 mM^−1^ cm^−1^). This method is specific for the native P450-domain, therefore the concentration of native fused PTDH is an estimation that might vary slightly from the actual concentration.

The concentration of unfused PTDH was determined using Waddell’s method (Waddell 1956) and the estimated extinction coefficient at 280 nm (ε_280 nm_ = 26.5 mM^−1^ cm^−1^).

### Activity determination and kinetic measurements

NADPH oxidation or NADP^+^ reduction rates were measured spectrophotometrically at 30 °C by following the absorption at 340 nm over 2–5 min (ε_340_ = 6.2 × 10^3^ M^−1^ cm^−1^) in a final volume of 500 μL in a quartz cuvette. For the investigation of the properties of the P450_BM3_ (fused or WT), typical reaction mixtures contained lauric acid as a substrate (2.5 mM from a 25 mM stock in ethanol/methanol (1:1)) and enzyme (100 nM) in Tris buffer (50 mM, pH 7.5). The reaction was started by the addition of NADPH (250 μM). For the investigation of the PTDH, the reaction mixture contained enzyme (100 nM) and phosphite as the substrate (5 mM). NADP^+^ was added to start the reaction (250 μM). For the determination of kinetic parameters or concentration-based activity, the concentration of the respective component was varied. All measurements were performed in triplicate. Kinetic parameters were calculated using the Michaelis-Menten equation as a function of the respective substrate concentration in GraphPad Prism 6.

### Biotransformations in 96-well plate format

Purified enzyme (0.5 μM), substrate (10 μM), NADPH or NADP^+^ (0.15 mM), and phosphite (1.5 mM) were mixed in Tris/HCl buffer (50 mM, pH 7.5, 100 μL) in 96-well round-bottomed plates. The reaction mixtures were incubated at 30 °C in a water bath or a plate shaker at 500 rpm. Samples (50 μL) were taken and mixed with 100% acetonitrile (100 μL). After centrifugation (4000 rpm, 20 min, 4 °C), the resulting supernatant (50 μL) was transferred into 40% acetonitrile in MilliQ water (150 μL) and stored at −20 °C prior to analysis by UPLC-MS.

### Biotransformations investigating cofactor recycling

Purified enzyme (1 μM), lauric acid (2.25 mM, 10% ethanol/methanol as final concentration), NADPH (50 μM), and phosphite (4 mM) were mixed in Tris/HCl buffer (50 mM, pH 7.5) in plastic falcon tubes (15 mL with 3 mL reaction mixture). For the three different set-ups—PTDH-P450_BM3_, PTDH + P450_BM3,_ and P450_BM3_—reaction mixtures without enzyme and without NADPH addition were the controls. The biotransformations were incubated (30 °C, 180 rpm) and samples for analysis in the molybdate assay and by GC-MS were taken in triplicate per time point (20 and 120 μL).

### Phosphate quantification via molybdate assay

Samples were taken from in vitro biotransformations to investigate cofactor recycling (20 μL). These were mixed with molybdate reagent (200 μL; 100 mM zinc acetate, 10 mM ammonium molybdate, and 0.1% SDS in MilliQ water, pH 5 (adjusted with HCl; stored in a PE bottle). Before an incubation (30 min, 30 °C), ascorbic acid (50 μL of a 10% *w*/*v* stock solution in MilliQ water, pH 5 (adjusted with NaOH)) was added. Measurements were performed in a Powerwave 96-well plate reader (Bio-TEK Instruments) at 850 and 700 nm. Phosphate standards containing potassium dihydroxy phosphate (0.1–5 mM) were used to prepare the calibration curve (Saheki et al. 1985; Dudek et al. 2013).

### GC-MS analysis

Samples from biotransformations investigating cofactor recycling (120 μL) were taken, and the conversions stopped in Eppendorf tubes prepared with NaCl (6 mg) and H_2_SO_4_ (50%, 10 μL). The reaction mixtures were extracted twice with twice the volume of MTBE/hexane (1:1); the internal standard, decanoic acid (1 mM in the final derivatization mixture) was added during the first extraction with the MTBE/hexane phase. The organic phases were collected and evaporated. Samples were resuspended in MTBE (60 μL), followed by the addition of 1% TMS in BSTFA (60 μL) and incubated at 75 °C for 30 min for derivatization. Tubes were sealed with parafilm during derivatization (Lalman and Bagley 2004; Scheps et al. 2013).

Measurements were performed on a HP-5 column ((5% Phenyl) methylpolysiloxan, 30 m × 0.25 mm × 0.25 μm), with an injection volume of 1 μL at 300 °C (injection and interface temperature) and helium as carrier gas. The method features a temperature gradient from 40 to 300 °C (10 °C/min and 10 min isothermal at 300 °C) with a solvent cut-off of 6 min, a split ratio of 30, and a flow of 1 mL min^−1^. The detection limit was approximately 4 μM. 12-hydroxylauric acid was used as a model product to estimate the response factor for monohydroxylated lauric acids. Derivatized capric acid, lauric acid, and 12-OH lauric acid elute after 10.6, 12.9, and 16.6 min, respectively. The derivatized products were identified via their fragmentation patterns. 11-hydroxylauric acid (2), 10-hydroxylauric acid (3), and 9-hydroxylauric acid (4) eluted at 18.5, 18.4, and 18.1 min, respectively.

### UPLC-MS analysis

Samples were analyzed on a Waters ACQUITY UPLC liquid chromatography system coupled to a Waters Synapt HDMS mass spectrometer equipped with an electrospray (ESI) ionization source. Samples taken during biotransformations were placed in a 96-well Nunc plate (injection volume 5–10 μL). Chromatograph separations were carried out on an ACQUITY UPLC BEH C18 column (130 Å, 1.7 μm × 2.1 mm × 100 mm; Waters, Milford, MA, USA) applying a flow rate of 0.5 mL min^−1^ at a column temperature of 45 °C. The mobile phase consisted of ultra-pure water supplemented with formic acid (0.1% *v*/*v*, phase A) and pure acetonitrile (phase B). The gradient applied for separation was 0.0–6.0 min (10–70% phase B), followed by a return to the initial mobile phase composition over 0.01 min.

The MS analysis was performed under the following conditions using the parameters stated. Positive electrospray ionization (ESI) conditions in V-mode. A generic method with two scan functions was used as follows: *m/z* 80–1000, cone voltage 20 V and 0.1 s scan time, the collision energy (CE) in *function 1* was 20 V and in *function 2* an energy ramp of 15–45 V was applied, the transfer cell CE is 12 V. Data was collected in centroid mode. Leucine-enkephalin was used as lock mass (*m/z* 556.2771) for internal calibration at a concentration of 250 pg mL^−1^ and a flow rate of 0.04 mL min^−1^. The MS data was processed in MetaboLynx 4.1 (Waters, Milford, MA, USA) using both the mass defect filter (MDF) and the dealkylation tool. The list of proposed metabolites was reviewed manually. A semiquantitative estimation of substrate conversion and metabolite formation were assessed by calculation of the fraction of the substrate peak area or a product peak area to the total related peak area detected (Kulig et al. 2015).

## Results

### Enzyme expression and isolation

The plasmid pCre2-PAMO described by Torres Pazmiño et al. was used as a template to create an expression plasmid for the production of P450_BM3_ fused to the C-terminus of a codon-optimized and stabilized PTDH which carries a His-tag at its N-terminus (Torres Pazmiño et al. 2009). Using this construct, six amino acids (SRSAAG) link the two proteins into one fusion protein of 157 kDa. For producing the P450_BM3_ as a reference for the non-fused enzyme, the P450_BM3_-encoding gene was cloned into a regular pBAD vector, resulting in expression of P450_BM3_ with a C-terminal His-tag (119 kDa) (Fig. S1). The PTDH was expressed from a pBAD vector with an N-terminal His-tag (Dudek et al. 2013).

The expression of PTDH-P450_BM3_ was optimized in terms of the type of *E. coli* host strain (Table S1), the expression temperature, and the concentration of arabinose added for induction (Fig. S2). Four *E. coli* strains with different features for recombinant protein expression were tested for soluble production of PTDH-P450_BM3_, namely *E. coli* TOP10, *E. coli* BL21(DE3), *E. coli* C43, and *E. coli* SHuffle. Expression of soluble and functional enzyme was determined by CO-difference spectroscopy. The most efficient strain for production of PTDH-P450_BM3_ was *E. coli* TOP10, while almost no expression was detected in *E. coli* BL21(DE3). To optimize soluble expression in *E. coli* TOP10, the temperature and concentration of inducer were varied; the best yield was obtained at 17 °C and induction with 0.02–0.2% arabinose. As judged by SDS-PAGE analysis, addition of δ-amino levulinic acid further improved expression. Recombinant production of PTDH-P450_BM3_ and P450_BM3_ using *E. coli* TOP10 in a culture volume of 50 mL yielded 9.5 mg PTDH-P450_BM3_ and 9.8 mg unfused P450_BM3_ in the cell-free extract after 42 h cultivation. This shows that the functional expression of both P450_BM3_ variants is very efficient.

**Fig. 1:**
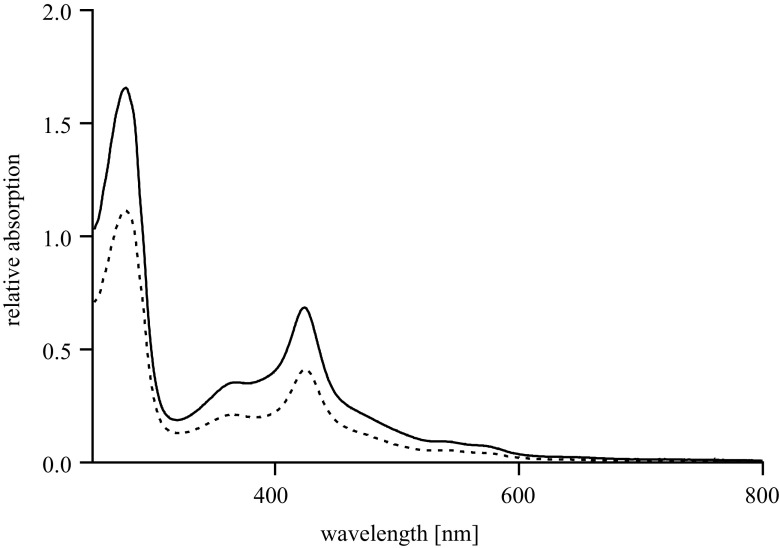
UV/Vis spectra of purified P450_BM3_ (*solid line*, 4.5 μM) and purified PTDH-P450_BM3_ (*dashed line*, 2.6 μM)

By exploiting the His-tag on both P450_BM3_ variants, both enzymes could be easily purified. The integrity of the purified P450_BM3_ and PTDH-P450_BM3_ was confirmed by the characteristic UV/Vis-spectrum (Fig. 1). The UV/Vis-spectral features of both proteins are nearly identical which confirms the incorporation of the essential heme and flavin cofactors.

**Scheme 2:**
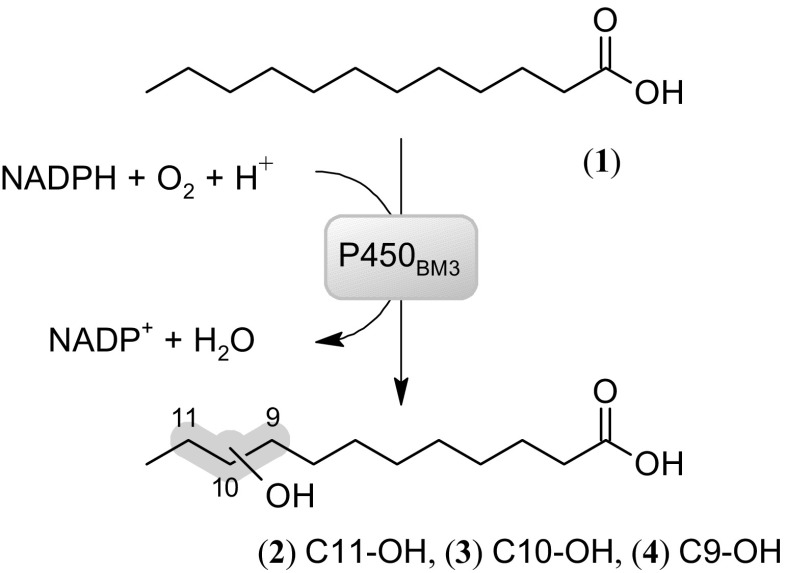
Model reaction catalyzed by P450_BM3_. NADPH is consumed during the monohydroxylation of lauric acid (**1**) in position 11, 10 and 9 (**2**,**3**,**4**)

**Fig. 2:**
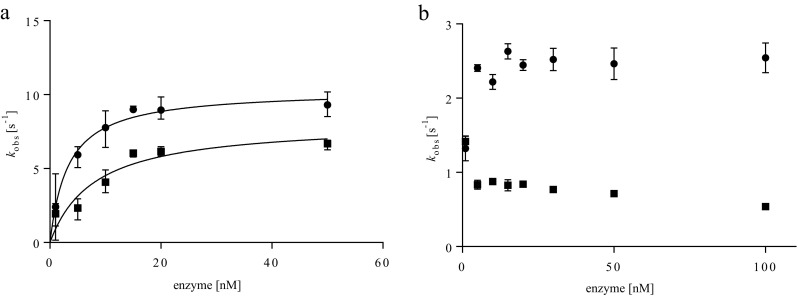
Enzyme concentration dependent activity of unfused P450_BM3_ (*squares*), unfused PTDH (*squares*), and PTDH-P450_BM3_ (*circles*). The graphs show the relation between enzyme concentration and the initial activities for **a** oxidation of 0.20 mM NADPH in the presence of 0.4 mM lauric acid as substrate at varying concentrations of either P450_BM3_ or PTDH-P450_BM3_ and **b** reduction of NADP^+^ in the presence of 2.0 mM phosphite as substrate at varying concentrations of either PTDH or PTDH-P450_BM3_. Concentrations of (PTDH)-P450_BM3_ were determined via CO-difference spectra, PTDH concentrations by Waddell’s method. Experiments were performed in triplicate

To verify whether the obtained enzymes were functional, monooxygenase and dehydrogenase activities were measured. For testing P450_BM3_ activity, conversion of lauric acid (1) by P450_BM3_ was analyzed. GC analysis revealed formation of monohydroxylation products in positions 11 (2), 10 (3), and 9 (4) in similar amounts (Scheme 2). By monitoring the rate of NADPH consumption using 50 nM enzyme, monooxygenase activities of 6.7 s^−1^ for P450_BM3_ and 8.9 s^−1^ for PTDH-P450_BM3_ were found, while uncoupling rates (defined as NADPH consumption in the absence of substrate) were determined as 0.22 s^−1^ for PTDH-P450_BM3_ and 0.19 s^−1^ for P450_BM3_. This shows that the fused enzyme displays a slightly higher monooxygenase activity (Fig. 2a). For PTDH activity, a similar trend was observed with a *k*_obs_ of 0.71 s^−1^ for the non-fused PTDH and 2.5 s^−1^ for the fused PTDH (Fig. 2b). These data show that PTDH-P450_BM3_ was fully functional, displaying both monooxygenase and dehydrogenase activities.

### Enzyme concentration dependent activity

Neeli et al. demonstrated that inter-monomer electron transfer is required for efficient hydroxylation of lauric acid and therefore the dimeric form of P450_BM3_ is the catalytically active variant (Neeli et al. 2005). To examine if the oligomerization behavior of the fused enzymes affected the reactivity of the fusion construct, the specific activities of the two non-fused enzymes and PTDH-P450_BM3_ were determined by following the consumption or generation of NADPH at different enzyme concentrations (1–50 nM for the P450 reaction, 1–100 nM for the PTDH reaction). The observed monooxygenase activity decreased sharply at enzyme concentrations below 10 nM. Hyperbolic fitting of *k*_obs_ values versus enzyme concentration gave apparent half-saturation concentrations of 8.8 ± 2.1 and 3.3 ± 0.9 nM for P450_BM3_ and PTDH-P450_BM3_, respectively (Fig. 2a). A similar enzyme concentration dependence was previously reported for native P450_BM3_ and is assumed to reflect the dissociation constant *K*_D_ for the dimerization of P450_BM3_, being active as a dimer (Neeli et al. 2005). The lower *K*_D_ for the fusion enzyme suggests that the fusion partner promotes oligomerization.

The rate of phosphite-dependent NADP^+^ reduction by PTDH (−P450_BM3_) was determined as a function of the concentration of the fused or non-fused PTDH (Fig. 2b). The *k*_obs_ for the PTDH reaction was relatively unaffected at enzyme concentrations of 5–100 nM. However, at an enzyme concentration of 1 nM, a decrease in the specific activity was observed for PTDH-P450_BM3_, while an increase was detected for the non-fused PTDH.

### Kinetic parameters of PTDH-P450_BM3_

The steady state kinetic parameters of PTDH-P450_BM3_ were determined and compared to those of the non-fused PTDH and P450_BM3_. For analyzing the P450 kinetic parameters, the lauric acid-dependent oxidation of NADPH was measured in the absence of phosphite for the fusion enzyme and for P450_BM3_ (Table 1). The phosphite-dependent reduction of NADP^+^ was measured for PTDH-P450_BM3_ and PTDH in the absence of lauric acid (Table 2). The kinetic properties of the fused enzyme were found to be in the same range as those of the separate enzymes. While the *K*_m_ values differed marginally, PTDH-P450_BM3_ displayed a higher *k*_cat_ for both enzyme domains (for PTDH activity, 1.9 s^−1^ for PTDH vs. 2.3 s^−1^ for PTDH-P450_BM3_; for P450 activity, 7.3 s^−1^ for P450_BM3_ vs. 8.0 s^−1^ for PTDH-P450_BM3_). These results confirm that the fused system is fully functional and that both domains are at least as efficient as their non-fused counterparts. When considering lauric acid, the PTDH showed a lower activity than the P450_BM3_ component. Therefore, the phosphite-dependent generation of NADPH should be the rate-limiting step during bioconversions. Yet, for other P450_BM3_ substrates, lower rates have been reported which makes PTDH a suitable NADPH regeneration partner.

**Table 1:** Kinetics of NADPH dependent lauric acid oxidation. Kinetic parameters of PTDH-P450_BM3_ and P450_BM3_ for NADPH and lauric acid were analyzed by following absorption of NADPH in a spectrophotometer at 340 nm. Specific activities were determined with 100 nM of the respective enzyme and varying concentrations of either NADPH or lauric acid. When fixed, NADPH had a concentration of 200 μM and lauric acid of 2.5 mM

	NADPH			Lauric acid		
	*k*_cat_ [s^−1^]	*K*_m_ [μM]	*k*_cat_/*K*_m_ [s^−1^ mM^−1^]	*k*_cat_ [s^−1^]	*K*_m_ [μM]	*k*_cat_/*K*_m_ [s^−1^ mM^−1^]
PTDH-P450_BM3_	6.9 ± 0.5	15.4 ± 2.3	448	8.0 ± 0.3	688 ± 78	11.6
P450_BM3_	6.0 ± 0.3	17.8 ± 2.7	337	7.3 ± 0.2	1427 ± 113	5.1

**Table 2:** Kinetics of phosphite-dependent cofactor regeneration. Kinetic parameters of PTDH-P450_BM3_ and PTDH for NADP^+^ and phosphite were analyzed by following absorption of NADPH in a spectrophotometer at 340 nm. Specific activities were determined with 100 nM of the respective enzyme and varying concentrations of either NADP^+^ or phosphite. When fixed, NADP^+^ had a concentration of 200 μM and phosphite of 2 mM

	NADP^+^			Phosphite		
	*k*_cat_ [s^−1^]	*K*_m_ [μM]	*k*_cat_/*K*_m_ [s^−1^ mM^−1^]	*k*_cat_ [s^−1^]	*K*_m_ [μM]	*k*_cat_/*K*_m_ [s^−1^ mM^−1^]
PTDH-P450_BM3_	2.10 ± 0.02	9.5 ± 0.5	221	2.30 ± 0.01	148 ± 4	15.5
PTDH	1.78 ± 0.02	3.9 ± 0.2	452	1.87 ± 0.02	201 ± 10	9.3

### Phosphite-driven drug conversion under screening conditions

The high catalytic activity makes P450_BM3_ an attractive biocatalyst for drug metabolism studies. Variants of the enzyme can be used for the production of human metabolites (Reinen et al. 2011). The application of PTDH- P450_BM3_ for exploring drug metabolism under screening conditions was investigated with omeprazole, a treatment for gastroesophageal reflux disease, and rosiglitazone, an antidiabetic drug. Both drugs are metabolized by P450_BM3_ (Whitehouse et al. 2012).

To investigate the functionality of the PTDH-P450_BM3_ system, conversion of both drugs was performed with NADPH or with phosphite plus NADP^+^ as electron source. Furthermore, conversion by the fusion enzyme was compared with conversions catalyzed by P450_BM3_. For these conversions, 10 μM of either omeprazole or rosiglitazone were incubated at 30 °C with purified P450_BM3_ or PTDH-P450_BM3_ in a final reaction volume of 100 μL. Samples were taken after 0 and 4 h and analyzed by UPLC-MS.

**Scheme 3:**
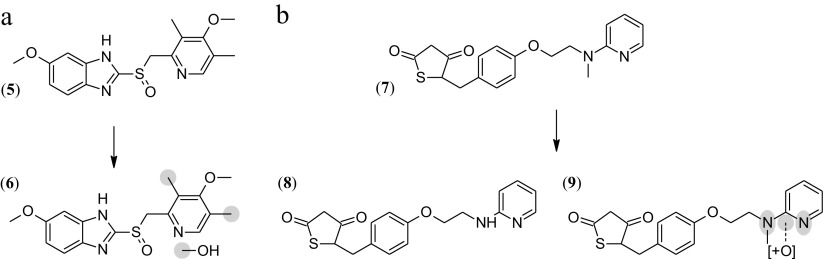
Conversions of **a** omeprazole (**5**) to its proposed monohydroxylation metabolite (**6**) and **b** rosiglitazone (**7**) and the proposed demethylation (**8**) and N-oxidation metabolites (**9**)

**Fig. 3:**
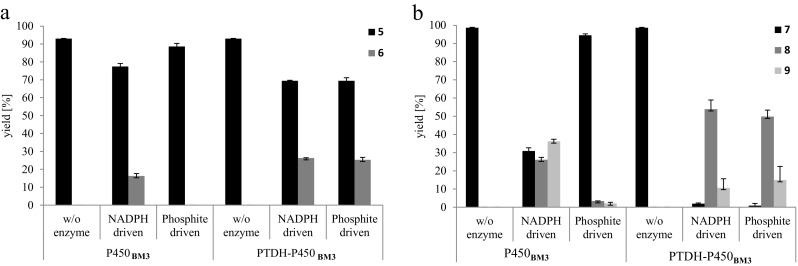
Conversions of **a** omeprazole and **b** rosiglitazone by P450_BM3_ and PTDH-P450_BM3_. Substrate was used at concentrations of 10 mM in a total volume of 100 mL. Reactions were carried out at 30 °C; reaction progress was measured after 4 h in comparison to 0 h samples by UPLC-MS. Phosphite-driven reactions contained phosphite and NADP^+^, whereas NADPH-driven reactions contained NADPH and phosphite. The yield of product and remaining substrate was determined as a percentage of the respective peak area to the total related peak area. Reactions were performed in duplicate (**5** = omeprazole, **6** = monohydroxylated omeprazole, **7** = rosiglitazone, **8** = demethylated rosiglitazone, **9** = monohydroxylated rosiglitazone)

Both enzyme variants produced a single metabolite when using omeprazole (5) as substrate. A monohydroxylated metabolite (6) was evident from a mass shift from the parent compound of 15.996 Da. Hydroxylation of one of the methyl groups of the 4-methoxy-3,5-dimethylpyridine moiety in omeprazole was proposed on the basis of fragmentation data (Scheme 3a). As expected, conversion with P450_BM3_ only occurred when NADPH was added as an electron source and not in reactions with phosphite and NADP^+^. Around 16% of the hydroxylated metabolite was formed in the NADPH-driven P450_BM3_ reaction (Fig. 3a). In the NADPH-driven reaction with the fusion enzyme, 25% of the same metabolite was formed. Reactions driven on phosphite as the sole electron donor showed similar behavior in terms of product formation. Under the tested conditions, the bifunctional enzyme was more efficient compared to non-fused P450_BM3_.

In reactions with rosiglitazone (MH^+^ 358.12; **7**) two main products were formed: a demethylated metabolite (MH^+^ 344.11; 8) and an N-oxide (MH^+^ 374.12; 9) (Scheme 3b) were proposed based on the fragmentation data. The unfused P450_BM3_ produced these metabolites in reactions with NADPH as electron donor with a yield of 26% for the demethylation and 36% for the N-oxidation product after 4 h. Very little conversion (<5%) occurred when NADP^+^ and phosphite were used. The ratio of demethylation vs. N-oxidation shifted when using PTDH-P450_BM3_; around 50% demethylation and 15% N-oxidation product were formed in reactions with the bifunctional enzyme (Fig. 3b). As observed for omeprazole, the total turnover was higher in reactions with the fusion enzyme. Furthermore, conversions with PTDH-P450_BM3_ did not show a significant difference when changing the electron source (NADPH or phosphite).

### Cofactor recycling

To establish the efficiency of the engineered PTDH-P450_BM3_ fusion enzyme with respect to cofactor recycling, conversions were analyzed in greater detail. Omeprazole and rosiglitazone are poorly soluble in aqueous solution and so lauric acid was used as a model substrate to investigate bioconversions with limited NADPH supply. Reactions were performed with the fused enzyme (PTDH-P450_BM3_), the non-fused parent proteins (PTDH + P450_BM3_), and P450_BM3_ without any cofactor recycling enzyme. Reaction mixtures consisted of 2.25 mM lauric acid, 4 mM phosphite, 50 μM NADPH, and 1.0 μM enzyme. As a consequence, conversions of lauric acid greater than 2.2% relied on cofactor regeneration by the PTDH. Conversions were monitored over time by taking samples for the analysis of phosphate production and the quantification of lauric acid and its metabolites.

Uncoupling was investigated by comparing the amount of phosphate formed, as an indicator of the amount of NADPH regenerated, and the amount of lauric acid oxidized. The concentration of phosphate was measured via a blue reaction product formed with molybdate (Saheki et al. 1985). Formation of phosphate which exceeded the production of hydroxylated lauric acids indicated uncoupling was occurring. Both the conversion of lauric acid and the amount of products formed were quantified after TMS derivatization by GC-MS analysis (Scheps et al. 2013). Three monohydroxylation products of lauric acid were identified via the characteristic elution and fragmentation patterns of their TMS derivatized products (Fig. S3 and Fig. S4) and were formed at varying concentrations. The products were identified as 11-hydroxylauric acid (2), 10-hydroxylauric acid (3), and 9-hydroxylauric acid (4).

**Fig. 4:**
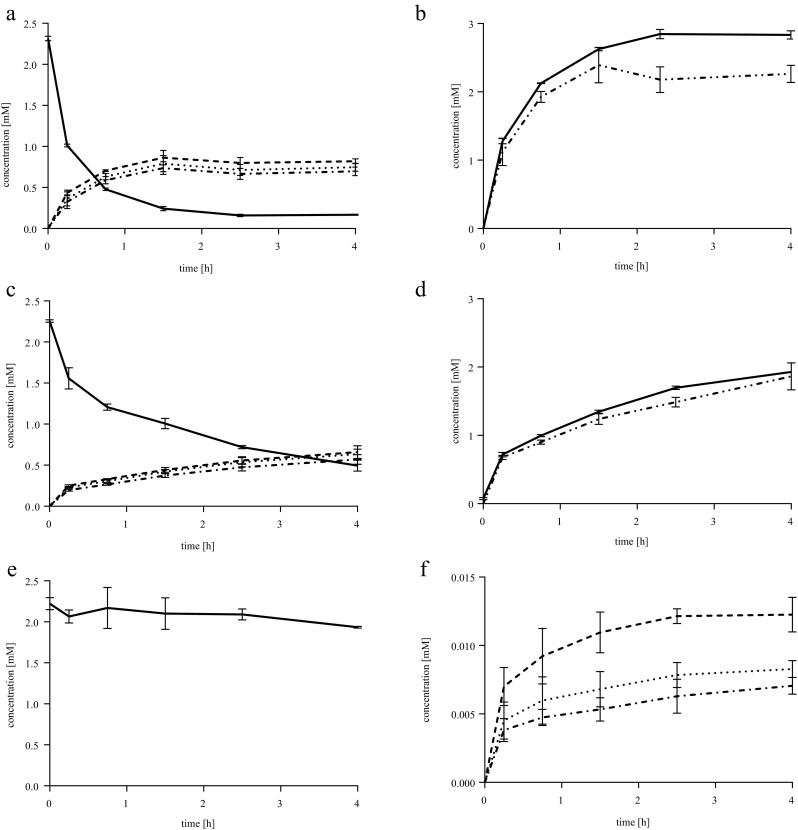
Conversions of lauric acid by different P450_BM3_ systems. All reactions contained 2.25 mM lauric acid, 4.0 mM phosphite, and 50 μM NADPH, thus making any product formation above 0.05 mM dependent on phosphite oxidation. **a**, **b** PTDH-P450_BM3_. **c**, **d** PTDH + P450_BM3_. **e**, **f** P450_BM3_. Samples were taken and analyzed by GC-MS to determine substrate depletion (**a**, **c**, and **e**; *solid line*) and product formation (**a**, **c**, and **f**) (9-hydroxylauric acid, *dotted line*; 10-hydroxylauric acid, *dotted dashed line*; 11-hydroxylauric acid, *dashed line*). In **b** and **d**, uncoupling was examined by following phosphate production measured with the molybdate assay (*solid lines*) and comparing it to total product formation determined by GC-MS (*double dotted dashed line*). For P450_BM3_ conversions, only a background of phosphate was detected (data not shown). All samples were measured in triplicate. Controls were reaction mixtures without enzyme and reaction mixtures without NADPH

Conversions of lauric acid by PTDH-P450_BM3_ reached completion after less than 2 h with a yield of 2.2 mM of combined monohydroxylation products in a ratio of 32.8% 9-hydroxylauric acid (4), 30.7% 10-hydroxylauric acid (3), and 36.7% 11-hydroxylauric acid (2). Excess phosphate, compared to the total amount of hydroxylated products formed, was produced mainly after conversion of lauric acid was complete. After 4 h, 25% more phosphate than product was formed, but during the first 1.5 h, only 10% phosphate production was uncoupled from substrate hydroxylation and uncoupling was still less earlier in the reaction (<5%). From the amount of monohydroxylated products formed, it was calculated back that phosphite supplied electrons for 44 turnovers of the NADPH cofactor. Even more turnovers of NADPH occurred, if the uncoupling is taken into consideration (Fig. 4a, b).

Conversions performed with non-fused PTDH and P450_BM3_ were less efficient than with the fusion enzyme (production of 1.6 mM monohydroxylated lauric acid after 2.5 h in comparison to 2.2 mM for the fusion enzyme). After 4 h, 1.86 mM of the combined hydroxylation products were formed with a similar ration between 9-, 10-, and 11-hydroxylauric acid as observed for the fused enzyme. The reaction did not reach completion after 4 h. Thus, phosphate production was still coupled to the product formation (approx. 6% uncoupling). In the time frame analyzed, at least 36 turnovers of the NADPH cofactor occurred (Fig. 4c, d).

The P450_BM3_ without cofactor regeneration system was not able to drive the reaction to a product yield near the concentration of cofactor added (only 27.6 μM hydroxylated product formed, with 1.94 mM of lauric acid remaining, when 50 μM NADPH were supplied). With 30.0% 9-hydroxylauric acid (4), 25.6% 10-hydroxylauric acid (3), and 44.4% 11-hydroxylauric acid (2), the latter was favored more than in the other two reactions (Fig. 4e, f). As expected, no significant phosphate was produced in reactions without PTDH (data not shown).

Interestingly, in controls without NADPH added to the reaction, PTDH-P450_BM3_ and PTDH + P450_BM3_ still formed small amounts of product (16.4 and 10.4 μM of total monohydroxylated products), whereas no monohydroxylated products were formed in the controls with P450_BM3_. Possibly, these conversions are driven by phosphite and minor amounts of NADPH and/or NADP^+^ co-purified with the enzyme.

## Discussion

While the natural fusion of the P450 oxygenase domain to the BMR domain in P450_BM3_ already makes this P450 a convenient system for oxidative biocatalysis, a NADPH regenerating system is still required for its efficient application. With the design of PTDH-P450_BM3,_ a fully self-sufficient system for phosphite-driven conversion of substrates by P450_BM3_ was engineered. It utilizes the reducing power of phosphite to generate NADPH from NADP^+^, which then donates electrons into the P450 reaction cycle for oxidation. This simplifies enzyme handling by providing the benefits of a single biocatalyst, without compromising the catalytic properties of either the PTDH or P450_BM3_.

Specific activity was fully retained at lower enzyme concentrations for PTDH-P450_BM3_ compared to P450_BM3_, indicating a higher affinity for oligomerization. An increase in the specific activity was observed for both enzymes in the fusion. Fogle et al. postulated that product release is the rate-limiting step for the variant stabilized and optimized for the conversion of NADP^+^ (referred to as 12x-PTDH, lacking mutation A176R compared to the variant fused to P450_BM3_) (Fogle and van der Donk 2007). The C-terminal modification of the PTDH might lead to structural changes that promote the release of NADPH from the active site. For the P450_BM3_, the product release and the electron transfer from the BMR to the heme domain are described as rate limiting (Whitehouse et al. 2012). Considering that the PTDH is N-terminally fused to the heme domain of P450_BM3_, a structural change that influences product release seems likely. Additionally, the assembly of the BMR domain of one P450_BM3_ with the heme domain of a second P450_BM3_ may be promoted by the fusion, and therefore the electron transfer may improve.

Phosphite-dependent conversion of omeprazole and rosiglitazone as exemplary drugs by PTDH-P450_BM3_ showed comparable turnover to NADPH-driven reactions. The total turnover was slightly higher than with conversions containing P450_BM3_ and NADPH in excess. This indicates that the fused enzyme is more efficient despite the observation that PTDH showed a lower activity than P450_BM3_ when testing the fused domains separately. This can be explained by the relatively low rate of turnover of these drugs by P450_BM3_. Butler et al. reported conversion of omeprazole by wild-type P450_BM3_ implementing glucose-6-phosphate dehydrogenase as a cofactor recycling system. In reaction mixtures with purified enzyme (0.1 μM) and the same concentration of substrate as used here (10 μM), less than 1% product formation was observed within 30 min. Higher conversions are described for mutants in the heme domain of P450_BM3_ (e.g., F87 V ∼50%) (Butler et al. 2013). In this study, we report a higher turnover for the wild-type P450_BM3_ without any cofactor recycling system (16%) and a slightly higher turnover for the fusion enzyme (25–26%). The higher yields are most likely caused by the higher enzyme concentrations that we employed and a prolonged reaction time. Clearly, it would be attractive to incorporate known mutations in P450_BM3_ in order to generate a powerful phosphite-driven PTDH-P450_BM3_ capable of generating drug metabolites.

The *p*-hydroxylation of the pyridine ring and N-demethylation of rosiglitazone are both major reactions when considering the metabolism of these drugs in humans while the N-oxidation is not listed as a relevant pathway (Cox et al. 2000). N-demethylation is mainly performed by the human P450 2C8 (Baldwin et al. 1999). Rosiglitazone conversions of up to 99% by mutants of P450_BM3_ using glucose-6-phosphate dehydrogenase for cofactor recycling have been described by Reinen et al. 2011. In conversions by PTDH-P450_BM3,_ less than 3% of rosiglitazone was detected in reactions after 4 h, suggesting that conversions by the fusion enzyme were comparably efficient. Integrating the mutations described by Reinen et al. might increase the total turnover slightly—ratios for demethylation and N-oxidation products were not described. Interestingly, we observed that the ratio of two formed metabolites from rosiglitazone depended on which P450_BM3_-based biocatalyst was used; native P450_BM3_ favored N-oxidation, whereas the fusion enzyme showed a strong preference for formation of one of the major human metabolites, the demethylated product. The N-terminal region of the unfused P450_BM3_ is described as one of the regions that are involved in the switch of conformation between the open and closed state (Ravichandran et al. 1993). We therefore assume that a fusion to the N-terminus might induce minor changes in the structure of the active site or the substrate access channel that influence regioselectivity.

With lauric acid as a model compound for conversions at higher substrate concentrations (2.25 mM), the fused enzyme proved superior to the non-fused system. Using PTDH-P450_BM3_, the conversion was complete within 2 h, while using non-fused P450_BM3_, the reaction did not reach completion within 4 h. In both cases, the reaction was driven far beyond the amount of NADPH added, while significant uncoupling of phosphate production from the formation of the monohydroxylated products only took place after the reaction had reached completion. The ratio of monohydroxylation products of lauric acid was in agreement with what was reported by Miura et al. for the NADPH-driven conversion by wild-type P450_BM3_ (Miura and Fulco 1975).

Watanabe et al. investigated a fusion of PTDH to the “PCNA-utilized protein complex of P450 and its two electron transfer-related proteins” (PUPPET) for the regeneration of NADH in conversions by P450cam (Watanabe et al. 2013). The PTDH-PUPPET fusion retained, similar to the PTDH-P450_BM3_ fusion, kinetic parameters comparable to the parent constructs, and the consumption of camphor was driven on phosphite and NAD^+^. The rate of NAD^+^ reduction by PTDH was lower than the rate of NADH oxidation by the P450, as we report for the PTDH-P450_BM3_ system. In both cases, cofactor reduction was assumed to be the rate-limiting step. In biotransformations with 1 mM D-camphor, 20 μM NAD^+^, and 10 mM phosphite, a turnover of the cofactor of 46 was reached, while here more than 50 turnovers based on phosphate production in conversions of lauric acid are described. Watanabe et al. achieved the same turnover in reactions with the non-fused PTDH after 8 h reaction time, suggesting that unfused PTDH and P450_BM3_ may reach the same turnover as the fused system over an extended incubation. While Watanabe et al. implement a NADH-dependent system, here the recycling of the more expensive NADPH is investigated. This demonstrates the capability of the PTDH to cooperate with different electron donor systems of P450s. Coupling to various P450s enhances the scope of substrate conversions that can be driven on phosphite.

A limiting factor in the conversions reported by Watanabe et al. was a loss of activity of the PTDH during incubations with phosphite. The use of a stabilized version of the PTDH for our studies might have prevented this effect for conversions by PTDH-P450_BM3_. The main limiting factor was the substrate solubility for rosiglitazone and omeprazole and substrate and/or product inhibition in case of lauric acid. It is expected that these bottlenecks can be overcome by enzyme immobilization, as described for the PTDH-PUPPET fusion and non-fused P450_BM3_ (Maurer et al. 2003; Weber et al. 2010; Lee et al. 2014; Tan et al. 2015).

Overall, these findings suggest that the designed fusion enzyme PTDH-P450_BM3_ is an efficient bifunctional biocatalyst with potential application in drug metabolism studies and for the production of fine chemicals.

## Electronic supplementary material

ESM 1 (PDF 282 kb)
