# Supplementary material for: P450BM3 fused to phosphite dehydrogenase allows phosphite-driven selective oxidations
Source: Appl Microbiol Biotechnol. 2016 Nov 29;101(6):2319–31. doi: 10.1007/s00253-016-7993-7 (PMC5320008; doi:10.1007/s00253-016-7993-7)
Supplement: Supplementary file 1 — (PDF 282 kb) [file 253_2016_7993_MOESM1_ESM.pdf]

Applied Microbiology and Biotechnology

$P450_{BM3}$ fused to phosphite dehydrogenase allows phosphite-driven selective oxidations

Nina Beyer[a], Justyna K. Kulig[b], Anette Bartsch[a], Martin A. Hayes[b], Dick B. Janssen[a], Marco W. Fraaije[a]

[a] Department of Biochemistry, University of Groningen, Groningen, The Netherlands
[phone: +31 50 363 4345; e-mail: m.w.fraaije@rug.nl]

[b] Cardiovascular and Metabolic Diseases, DMPK, Innovative Medicines and Early Development Biotech Unit, AstraZeneca, Sweden

Corresponding author:

Marco W. Fraaije, Department of Biochemistry, Groningen Biomolecular Sciences and Biotechnology Institute University of Groningen, Nijenborgh 4, 9747 AG Groningen, The Netherlands
Tel. 0031-50-3634345, e-mail: m.w.fraaije@rug.nl

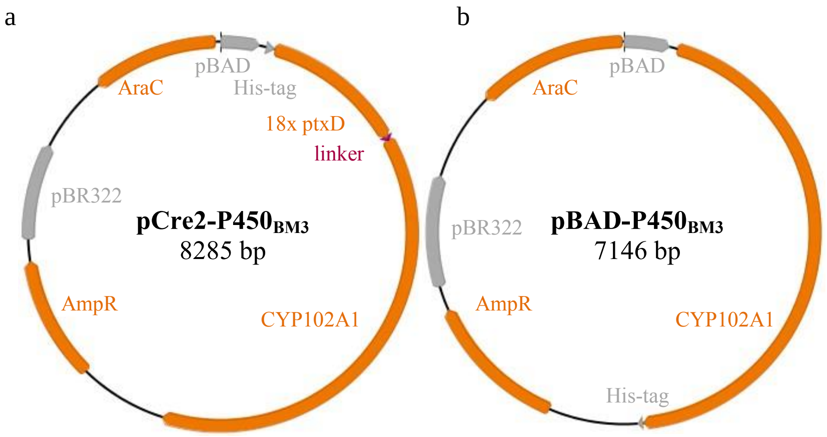

Fig S1 Plasmid maps of the a) synthetic construct pCre2-P450$_{BM3}$ for the production of the fully self-sufficient PTDH-P450$_{BM3}$, in comparison to b) pBAD-P450$_{BM3}$ as expression vector for P450$_{BM3}$ WT in *E. coli*.

Table S1 Qualitative analysis of the level of PTDH-P450$_{BM3}$ in the cell free extract after expression from a pBAD vector in different *E. coli* strains under the same conditions, determined by CO-difference spectra.

***E. coli* strain**	**Expression level** [mg/50 mL culture]
Top10	4.7
BL21 DE3	0.0
C43	0.8
SHuffle	0.7

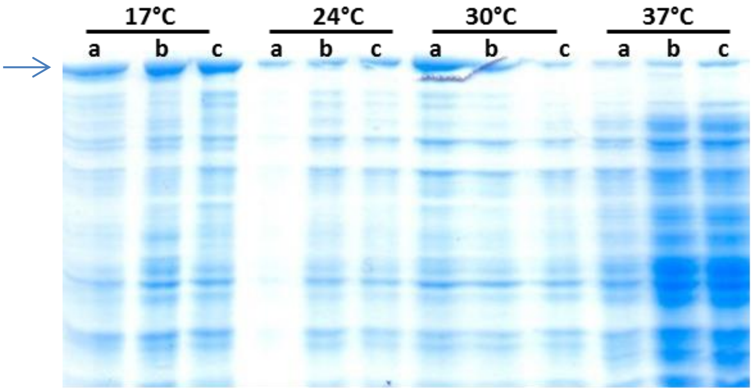

Fig S2 Expression optimization of PTDH-P450$_{BM3}$ by varying expression temperature and varying arabinose concentration. Soluble fraction obtained after expression in *E. coli* TOP10 at different temperatures and arabinose concentrations a = 0.002%, b = 0.02% and c = 0.2%.. Proteins were separated in a 12% SDS-Page gel. The protein band of PTDH-P450$_{BM3}$ is highlighted with an arrow.

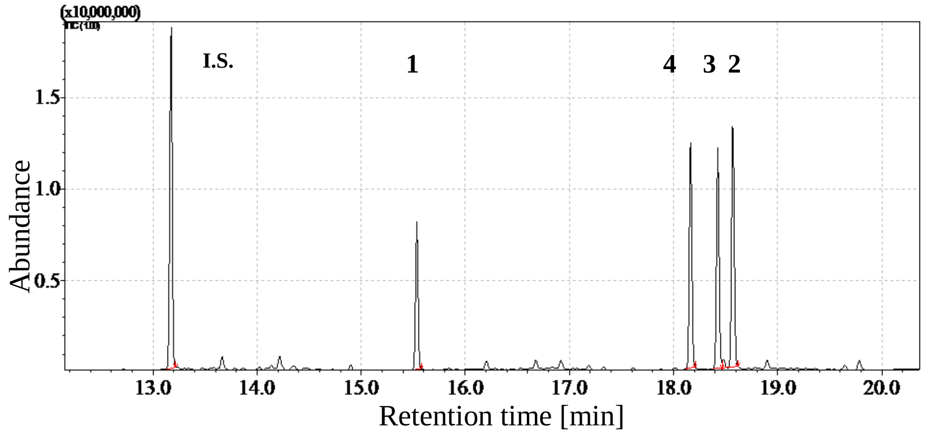

Fig S3 Representative chromatogram of TMS-derivatized lauric acid and the hydroxylated products from conversions with P450$_{BM3}$. For quantitative comparison all peak areas were normalized relative to the peak area of capric acid, the internal standard (I.S.). The response factor of the monohydroxylated TMS-derivatized products was estimated from TMS-derivatized 12-hydroxylauric acid.

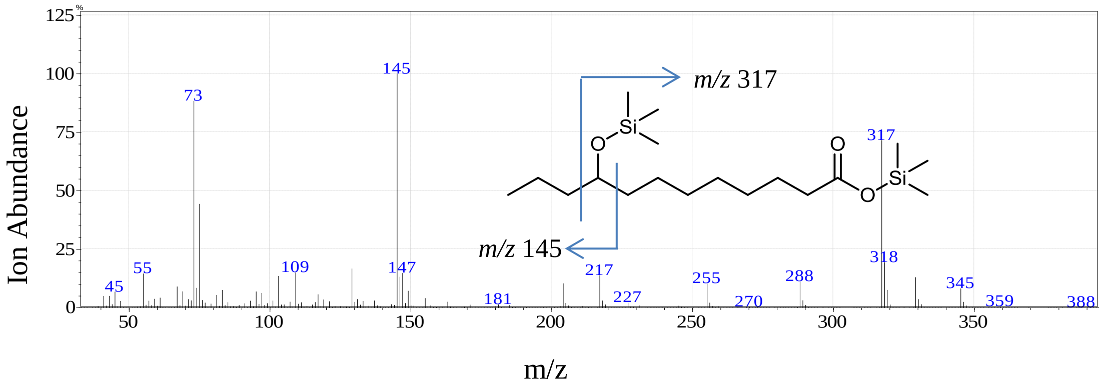

Fig S4 Representative fragmentation spectra for TMS derivatized 9-hydroxylauric acid. MS fragments correspond to the GC-MS peak at 18.1 min (TMS derivatized **4**).
